# Duplication of 17q11.2 and Features of Albright Hereditary Osteodystrophy Secondary to Methylation Defects within the GNAS Cluster: Coincidence or Causal?

**DOI:** 10.1155/2013/764152

**Published:** 2013-01-14

**Authors:** M. White, J. Conroy, H. Bullman, M. Lever, E. Daly, D. R. Betts, D. Cody, John A. Crolla, S. A. Lynch

**Affiliations:** ^1^Department of Endocrinology, Our Lady's Children's Hospital, Crumlin, Dublin 12, Ireland; ^2^Medical Genetics Research Group, Health Sciences Centre, University College Dublin, Belfield, Dublin 4, Ireland; ^3^Wessex Regional Genetics Laboratory, Salisbury District Hospital, Salisbury WILTS SP2 8BJ, UK; ^4^Department of Paediatrics, Midland Regional Hospital, Portlaoise, Co Laois, Ireland; ^5^National Centre for Medical Genetics, Our Lady's Children's Hospital, Crumlin, Dublin 12, Ireland; ^6^Cytogenetics, National Genetics Reference Laboratory (NGRL), Wessex, UK; ^7^Salisbury District Hospital, Salisbury SP2 8BJ, UK

## Abstract

We report a case of Albright hereditary osteodystrophy (AHO) in a three-year-old girl with a microduplication at 17q11.2. The child developed obesity within the first 6 months of life. A diagnosis of Albright was made at age 2 years when biochemical evidence of parathyroid resistance was found. No mutations were identified in guanine nucleotide-binding protein G (s) subunit alpha (GNAS1). Subsequent investigations revealed methylation disturbance at GNAS1A, neuroendocrine secretory protein antisense (NESPAS) and neuroendocrine secretory protein 55 (NESP55) confirming a diagnosis of pseudohypothyroidism type 1B. A deletion of NESP55 and uniparental disomy chromosome 20 were excluded which suggested that the features of AHO arose through a purely epigenetic mechanism. Further investigation revealed a *de novo* microduplication at 17q11.2 encompassing the neurofibromatosis type 1 (NF1) gene. The combination of two rare *de novo* events in the same child raises the possibility that duplication of a gene within the 17q11.2 region may have triggered abnormal methylation in the GNAS cluster region on chromosome 20.

## 1. Clinical Summary 

The child, a girl, was the third child born to healthy non consanguineous parents at 38 weeks of gestation by Elective Lower Segment Caesarean Section weighing 3.01 kilograms. There were no neonatal issues apart from mild jaundice that did not require phototherapy treatment, and family history was noncontributory. At 3 months of age she was noted to be on the 99.9th centile for height and weight, which was to remain an ongoing concern, and she developed a large appetite with little satiety from early infancy. Development was initially felt to be mildly delayed globally, and at 18 months she was behind in gross motor and communication development. However, at 2.5 years her development was reported as being within normal limits.

The child had been attending her local paediatrician for weight management and dietetic input. Two separate TSH measurements were elevated with a normal Free T4 on both occasions. This prompted referral to a tertiary endocrinology service at 2 years of age for endocrinology assessment of elevated TSH in the context of obesity. 

At the initial endocrinology review, her weight was 20 kg (>99.6th centile), height was 93 cm (>99.6th centile), and body mass index (BMI) was 23.2 (>99.6th centile for age and gender). Clinical examination revealed a round facies, a flat occiput and central obesity. Phenotypic appearances suggested a possible diagnosis of AHO, without any evidence of the skeletal manifestations, either clinically or radiologically. Bone age was appropriate for chronological age. Serial serum electrolytes have all shown normal calcium and phosphate, with persistently elevated parathyroid hormone (PTH) and abnormal thyroid function tests (Table 1). Mild primary hypothyroidism was suspected and confirmed on a TSH stimulation test (Table 2).

## 2. Genetic Analysis

Chromosome analysis revealed a normal female karyotype, and Prader-Willi syndrome was excluded by FISH analysis. Subteleomere FISH analysis on chromosome 2q37 (D2S447, Vysis) was performed because of the clinical suspicion of AHO. This revealed a diminished signal on one chromosome 2 homologue, thereby consistent with a partial deletion of this probe. Subsequent subtelomere FISH was also performed with a dJ1011017 (Cytocell) probe, which was shown to be fully deleted. However, this latter locus is known to be polymorphic and is deleted in approximately 5% of the population. Subsequent array testing suggested this deletion to represent a common copy number variant of 67 kb in size [2 : 242579273-242646571-NCBI build 36].

**Figure 1:**
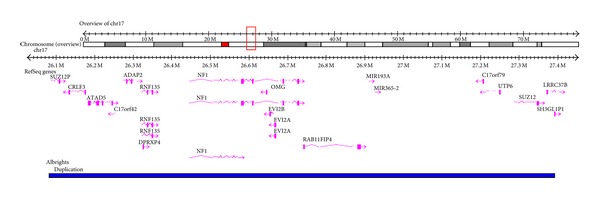
Array deletion map of area on 17q11.2 with deleted genes listed.

Array comparative genomic hybridisation (Array CGH) and confirmatory multiplex ligation-dependent probe amplification (MLPA) identified a 1.3 Mb duplication involving the region 17q11.2 (Figure 1, chr17: 26085852-27391268, NCBI build 36). This results in the duplication of 11 RefSeq genes and 2 miRNAs. One of the genes duplicated is the neurofibromatosis Type 1 (NF1) gene. FISH analysis confirmed this as a 17q11.2 duplication. This may be important in the context of learning difficulties reported by the patient. Parental studies (FISH) were normal confirming the *de novo* nature of the duplication.

Mutation analysis in the *GNAS1* gene identified no mutations; array and molecular analysis of this gene was normal. Methylation sensitive PCR (MSPCR) analysis *of *the *GNAS1A, NESPAS, *and* NESP55* differentially methylated regions (DMRs) was performed using bisulphite treated DNS prepared using the EZ methylation kit (Zymo Research). This demonstrated a significant loss of the maternal methylation pattern at all three sites, thus confirming a diagnosis of PHP-1B. Analysis of a repeat sample confirmed these results. In some patients methylation changes at multiple *GNAS* loci have been associated with microdeletions of NESP55 [1]. No such deletion was detected at this locus using long range PCR. Microsatellite analysis demonstrated both parental alleles at 5 loci flanking the *GNAS* locus, thus excluding uniparental disomy for chromosome 20 (UPD20).

Exclusion of UPD20 and an *NESP55* or other 20q13 deletion (array CGH did not detect a chromosome 20q13 deletion) suggested that the cause of the abnormal methylation pattern at the *GNAS* locus was purely epigenetic and the patient might possibly have had methylation disturbances at other loci. Multiple methylation abnormalities have been shown in patients with transient neonatal diabetes mellitus [2] and Beckwith-Wiedemann syndrome (BWS) [3]. Further methylation analysis was undertaken at 7 imprinted regions (*MEST, DLK1, SNRPN, GRB10, PEG3*, genes at the TND region, and the BWS genes) using published methods [2]. No imprinting disturbances were identified (data not shown). 

## 3. Discussion

This case report highlights the difficulty in the interpretation and explanation of abnormal results on increasingly complex genetic testing. We report a case of clinical AHO-type features, with a methylation abnormality in keeping with PHP-1b and microduplication in the NF1 region.

 A diagnosis of PHP-1b was made in the described patient due to the presence of biochemical PTH resistance despite the absence of skeletal manifestations of AHO.

The complex phenotype caused by duplication of 17q11.2 is unclear as there are few reported cases in the literature. The region encompasses the NF1 gene, and whilst deletions of this region have been extensively reported, duplication cases are poorly reported. Grisart et al. reported a single family with seven cases of NF1 microduplication in a two-generation family [4]. Five of the seven affected family members had mild-to-moderate developmental delay. Apart from early onset male patterned balding and dental enamel anomalies, there were no other striking phenotypic findings, and the pathological significance of the NF1 duplication remains unclear. Mutations in and deletions of the RNF135 gene have been reported in a number of patients, who share common features of increased postnatal growth (defined as height and/or head circumference at least 2 standard deviations above the mean) [5]. The Decipher website (http://decipher.sanger.ac.uk/) lists 2 microduplications within this region with corresponding phenotypic descriptions 253572 & 254309 and three other inherited duplications with no recorded phenotype. 

The report of a chromosome 17q11.2 duplication and an imprinting defect on chromosome 20q13.32 presents an intriguing question. One of the duplicated genes is *SUZ12,* which in, mammals, forms part of the polycomb repressive complex 2 (PRC2) core subunits in addition to Eed, Ezh2, and RbAp48 [6]. The mechanism through which PRC2 is recruited to chromatin is unclear. Recent publications have been highlighting the potential role of RNAs in PRC2 function. A study by Zhao et al. catalogued a genomewide pool of PRC2-interacting RNAs in embryonic stem cells [7]. One of the imprinted regions identified by this analysis was an antisense RNA from the primary imprinting control regions that is thought to regulate the NESP/GNAS cluster [7–9].

The clinical presentation of AHO is variable. The causality of the two identified molecular abnormalities is unclear in this case, and any association may be purely speculative, but the heterogeneity of AHO does not dilute the possibility of a link between the 17q11.2 and the clinical features in this case, and it may be worth considering testing other cases of NF1 microduplication for PTH levels.

## Figures and Tables

**Table 1:** Serial biochemistry results.

Date	Calcium (nmol/L)*	Alkaline phosphatase (U/L)^#^	Phosphate (mmol/L)^†^	T4 (pmol/L)^‡^	TSH (mIU/L)**	PTH (ng/L)^##^
17/7/2009	2.51	**431**	1.81	N/A	N/A	**229.1**
9/12/2009	2.34	**400**	N/A	13.1	**6.76**	**400.6**
12/1/2010	2.51	**390**	1.9	12.1	**8.21**	**334.6**
11/5/2010	2.49	**422**	2.18	11.7	**10.8**	**238.9**

Abnormal results in bold for emphasis.

Normal ranges: *Calcium 2.15–2.65 nmol/L, ^#^Alkaline phosphatase 45–315 U/L, ^†^Phosphate 1.16–2.1 mmol/L, ^‡^Thyroxine 10–22 pmol/L, **Thyroid Stimulating Hormone 0.1–5.0 mIU/L, and ^##^Parathyroid Hormone 11–35 ng/L.

**Table 2:** Thyrotropin-releasing hormone stimulation test.

Time (minutes)	TSH (mIU/L)	Free T4 (pmol/L)
0	8.74	17
20	49.2	
30	43.2	
60	19.6	

This TRH stimulation test shows an elevated TSH at baseline, with a corresponding normal T4 level, which outrules a TSH resistance. The brisk TSH response outrules thyroid Hormone releasing hormone (THRH) resistance.
